# Deep Pelvic Endometriosis

**DOI:** 10.5334/jbsr.2533

**Published:** 2021-09-14

**Authors:** Kelly Di Dier, Adelard De Backer

**Affiliations:** 1 AZ Sint-Lucas, BE

**Keywords:** gynaecology, urogenital radiology, endometriosis, deep pelvic endometriosis, MRI

## Abstract

**Teaching Point:** Magnetic resonance imaging allows adequate evaluation of the location, size and subperitoneal lesion extension of deep pelvic endometriosis, providing key information for both the diagnosis and treatment planning.

## Case

A 22-year-old female has had dysmenorrhea and irregular menstruation since her menarche. Transvaginal ultrasound showed a hypoechoic irregular mass at the posterior vaginal fornix.

**Figure 1:**
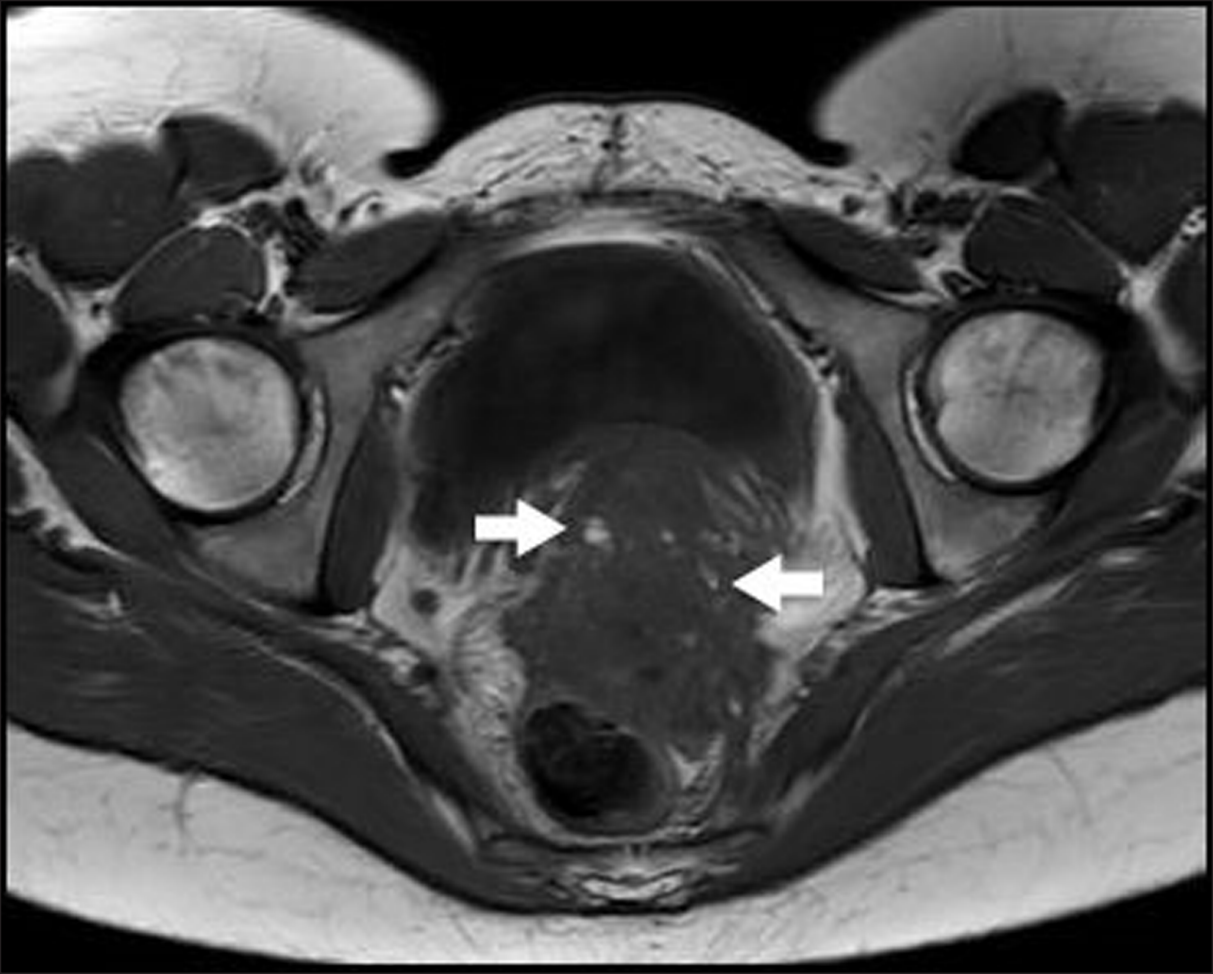


**Figure 2:**
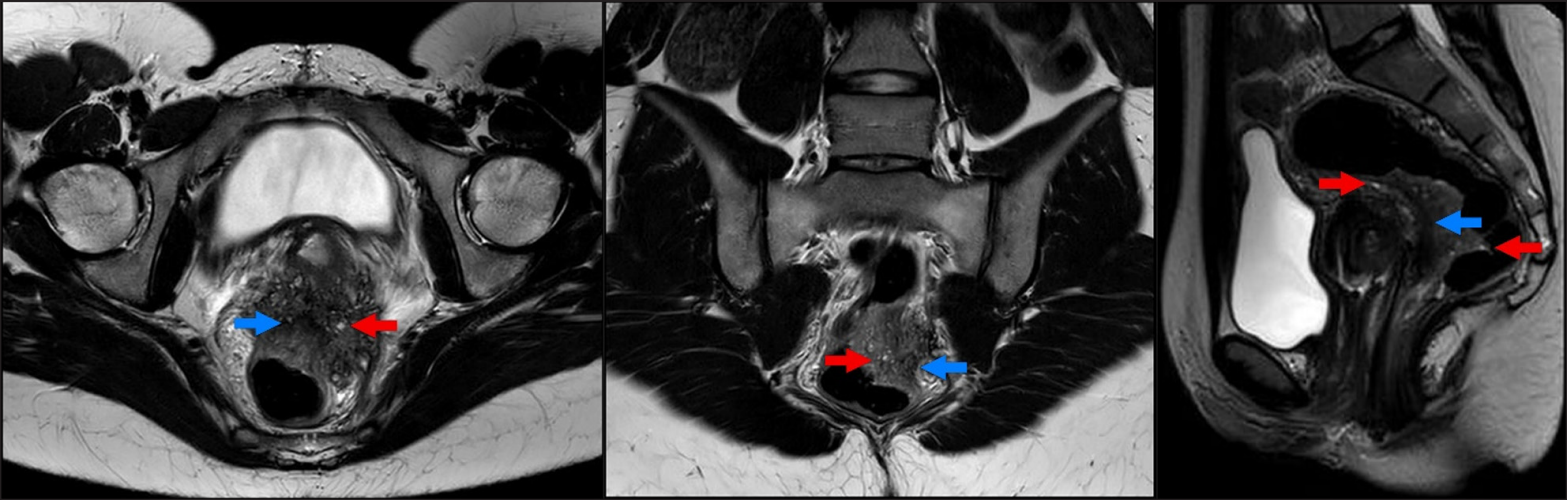


**Figure 3:**
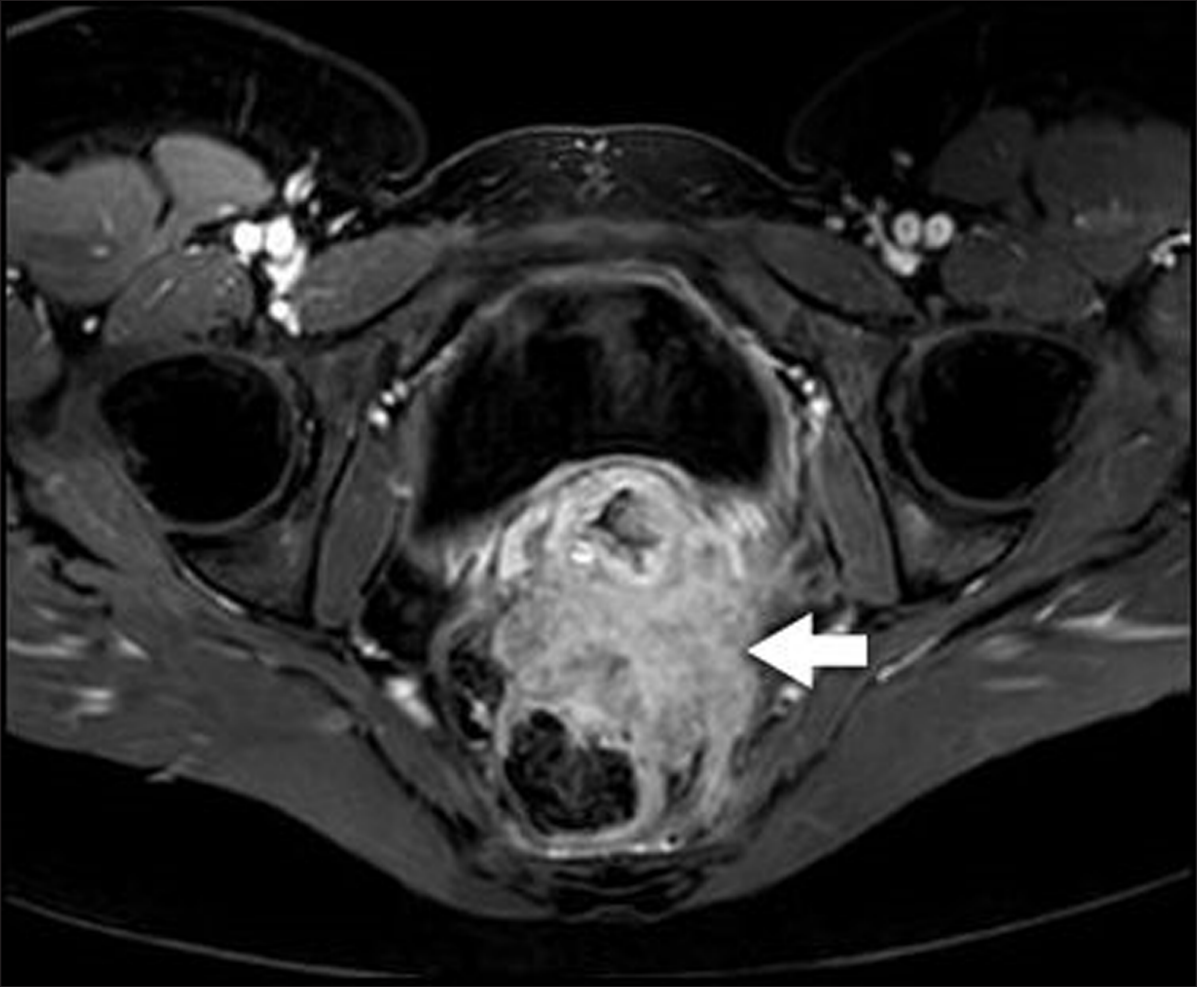


Pelvic magnetic resonance imaging (MRI) confirmed findings consistent with deep pelvic endometriosis (DPE). There was an irregularly delineated mass in the rectouterine pouch extending in the rectovaginal septum and the anterior rectal wall. Thickening of the mesorectal fascia and partial obliteration of the perirectal fat were noted. On T1-weighted images, the mass was a low-to-intermediate in signal intensity (SI), with punctate areas of hyperintensity (***Figure 1***, arrows). T2-weighted images demonstrated small areas of higher SI (***Figure 2***, red arrows), delineated by areas of low SI (***Figure 2***, blue arrows). After intravenous administration of Gadolinium, fat-suppressed T1-weighted images demonstrated a significant enhancement (***Figure 3***, arrow).

## Comment

DPE is a pathology in which functional endometrial tissue is present outside the uterus. Common locations are the ovaries, pelvic subperitoneal space, intestinal tract, and urinary system. MRI findings may vary from small implants, solid deep lesions (preferably located in the pouch of Douglas) and visceral endometriosis involving the bladder and rectal wall.

Clinical symptoms, if present, may include severe pelvic pain, dysmenorrhea, dyspareunia, dyschezia, urinary symptoms, and infertility [1].

DPE can be diagnosed on physical examination and transvaginal ultrasound. However, detailed evaluation of disease extension is difficult. Rectal ultrasound allows evaluation of rectal wall infiltration but shows poor penetration. DPE may be overlooked during exploratory laparoscopy due to adhesions or subperitoneal localisation. MRI allows accurate diagnosis and evaluation of the location, size, and extension into surrounding tissues of DPE [1].

On T1-weighted MRI, small areas of high SI may be present as in our case, representing foci of haemorrhage. On T2-weighted images, low SI may correspond to fibrotic tissue. As in our case, DPE may show heterogeneously higher T2-weighted SI due to a large proportion of glandular tissue which subsequently enhance after contrast administration, contrary to necrosis and haemorrhage [1]. 

When conservative therapy fails, radical surgery is the standard therapeutic approach. An accurate evaluation of the extension of the lesion is crucial to obtain complete excision and to prevent postoperative recurrence [1].

## References

[1] Del Frate C, Girometti R, Pittino M, Del Frate G, Bazzocchi M, Zuiani C. Deep retroperitoneal pelvic endometriosis: MR imaging appearance with laparoscopic correlation. Radiographics. 2006; 26(6): 1705–1718. DOI: 10.1148/rg.266065048 17102045

